# Multiple primary malignancies: synchronous urothelial carcinoma of the bladder and adenocarcinoma of the colon

**DOI:** 10.1590/0100-3984.2015.0114

**Published:** 2017

**Authors:** Rodolfo Mendes Queiroz, Daniel Roque, Eduardo Miguel Febronio

**Affiliations:** 1 Documenta - Hospital São Francisco, Ribeirão Preto, SP, Brazil.

Dear Editor,

A 75-year-old White male presented with a three-month history of pain in the left hypochondrium. The patient also reported experiencing an episode of gross hematuria six months prior. He had quit smoking 20 years prior, having previously smoked 30 cigarettes/day for 30 years. He had also undergone surgery for a gastric ulcer 20 years prior. He reported no other comorbidities.

**Figure 1:**
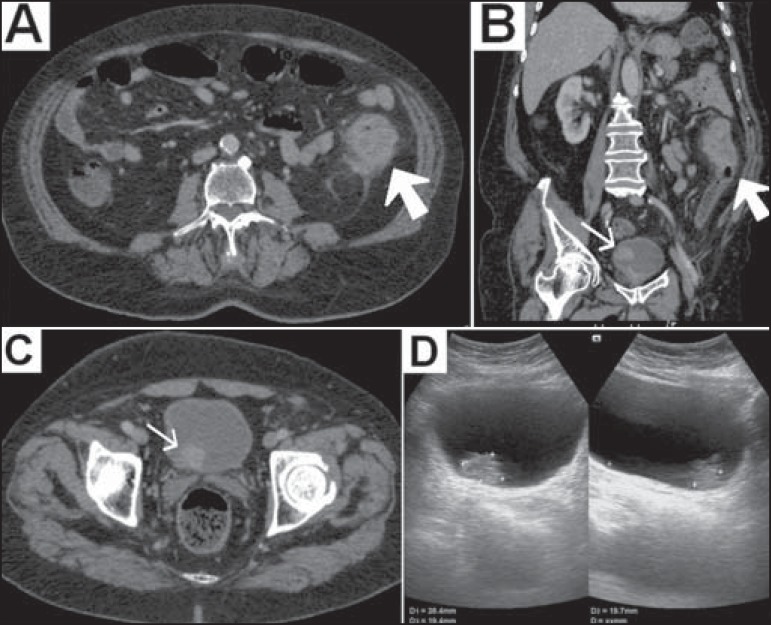
**A,B:** Computed tomography scan of the abdomen, obtained in the portal phase after intravenous administration of contrast medium, in an axial view (**A**) and oblique coronal reconstruction (**B**), showing a solid, irregular, concentric mass, which was expansive and stenotic, in the descending colon, presenting heterogeneous enhancement, together with increased density of the adjacent fat tissue (large arrow). Note also the vegetative lesion, with irregular borders and showing contrast enhancement (small arrow in **B**). **C:** Axial computed tomography slice, obtained in the portal phase after intravenous administration of iodinated contrast medium, showing the vegetative lesion, with irregular borders, located in the right posterolateral wall of the bladder (arrow). **D:** Abdominal ultrasound, confirming the lesion in the bladder wall.

Computed tomography of the abdomen showed a solid, irregular, concentric mass, which was expansive and stenotic, in the middle third of the descending colon (Figures 1A and 1B). The mass showed heterogeneous uptake of the intravenous iodinated contrast medium and increased density of adjacent fat tissue, suggesting that it had expanded through the serosa. In addition, a vegetative lesion, with irregular borders and showing contrast enhancement, was observed in the right posterolateral wall of the bladder (Figures 1B and 1C).

Colonoscopy with biopsy of the intestinal mass led to a histological diagnosis of moderately differentiated adenocarcinoma of the colon, and the patient was therefore submitted to segmental colectomy with colostomy. The anatomopathological study revealed a hard, annular tumor, which was ulcerative and vegetative, infiltrating the intestinal wall and surrounding fat, thus confirming the result of the microscopy study of the biopsy. Subsequently, ultrasound of the urinary tract confirmed bladder nodulation (Figure 1D), with no perceptible flow on color Doppler. Complete transurethral resection of the nodulation was performed, and histopathological analysis of the resected specimen led to a diagnosis of superficial low-grade papillary urothelial carcinoma (World Health Organization grade I). A subsequent computed tomography scan of the abdomen and pelvis, for staging, showed no suspicious lesions. The final diagnosis was multiple, synchronous primary malignancies, probably secondary to smoking.

Colon cancer is the fourth most common malignancy in men, accounting for 90% of the cases that occur after the fifth decade of life, adenocarcinoma being the most common type^(1)^.

In 5-10% of cases, adenocarcinoma is associated with hereditary syndromes (e.g., familial adenomatous polyposis, hereditary non-polypoid colorectal cancer, etc.), especially in young adults^(1)^. It is related to obesity, a sedentary lifestyle, a diet low in fiber, and inflammatory bowel diseases^(1-4)^. Smoking and alcoholism can also play roles^(2-4)^.

Bladder cancer, which is the most common type of malignant neoplasia of the urinary tract, affects individuals 55-60 years of age, 75-80% of whom are men, urothelial carcinoma being the predominant form^(5,6)^. Urothelial carcinoma can be multifocal/multicentric, can occur in the upper or lower urinary tract, and is often recurrent^(5)^. Smoking is implicated in 50-65% of all cases in men and in 20-30% of all cases in women^(4)^. Other, less common causes include chemotherapy, exposure to aromatic or heterocyclic amines, radiotherapy, and chronic infection^(2,4-6)^.

Multiple primary malignancies are defined as those that are confirmed, independent, and of non-metastatic origin^(7)^. They are classified as synchronous if they are identified within the first six months after the appearance of the first lesion or as metachronous if they are identified thereafter^(7)^.

The overall prevalence of multiple primary malignancies is 0.7-11.7%, increasing proportionally with patient age^(2,3,7,8)^. It is estimated that 75% of cases occur in individuals over 50 years of age^(7)^. These values are on the rise due to the effectiveness of treatments, the variety of therapeutic techniques now available, the improvement of diagnostic methods, the increased longevity of the population, and contemporary lifestyles^(3,7)^. Hayat et al.^(2)^ reported a probability of developing a second malignancy, depending on the primary tumors diagnosed, ranging from 1% (history of hepatic neoplasia) to 16% (previous bladder tumors)^(2)^. Braisch et al.^(4)^ observed that 1.2-2.5% of cancer patients who were smokers developed another distinct malignant lesion within the first year of follow-up.

In smokers, multiple primary malignancies can affect several organs, notably the lungs, upper aerodigestive tract, and kidneys, as well as the upper and lower urinary tract. Other potential sites include the thyroid gland, stomach, colon, rectum, and pancreas^(4,6,8)^.
